# 10399 DNA-PK(cs) Regulates Stability of Egr1 During T Cell Activation

**DOI:** 10.1017/cts.2021.420

**Published:** 2021-03-30

**Authors:** Zachary Waldrip, David Harrison, Marie Burdine, Lyle Burdine

**Affiliations:** University of Arkansas for Medical Sciences, Arkansas Children’s Research Institute

## Abstract

ABSTRACT IMPACT: This work provides supporting evidence for the development of a novel immunosuppression therapy for transplant patients. OBJECTIVES/GOALS: Our laboratory reported that inhibition of the kinase DNA-PK(cs) in mice delays allogeneic graft rejection in part by mitigating the induction of certain cytokines. We hypothesized that this was due to an inhibition of intracellular signaling programs in T cells and designed studies to identify the mechanism(s) by which this occurs. METHODS/STUDY POPULATION: The immortalized Jurkat T cell line was used to evaluate the effect of the DNA-PK(cs) inhibitor NU7441 on T cell activation by PMA/Ionomycin or PMA/PHA. Mouse primary splenocytes also were used to demonstrate the universality and reproducibility of our observations. Initially, protein mass spectrometry of lysates from untreated and NU7441-treated Jurkat cells identified proteins of interest regulated by DNA-PK(cs) that play a role in T cell activation and cytokine production. CRISPR genome editing was used to validate a potential downstream target of DNA-PK(cs). Western blot, ELISA, and flow cytometry were used to document changes in protein levels with respect to treatments. RESULTS/ANTICIPATED RESULTS: We observed that expression of the transcription factor Egr1 was highly induced after activation but attenuated after treatment with NU7441 in both Jurkat T cells and mouse splenocytes. Phosphorylated serine 301 of Egr1 was identified by mass spectrometry in stimulated cells and fits the kinase consensus sequence for DNA-PK(cs). Both an endogenous CRISPR-generated serine 301 to alanine mutant and expression of a plasmid-based S301A mutant resulted in an unstable form of Egr1 that was barely detectable. In contrast, expression of either a S301 to D or E phospho-mimetic mutant resulted in a stable form of the protein detectable by Western blot. Further evaluation of these mutants and Egr1 phosphorylation is underway to determine the mechanism by which DNA-PK(cs) kinase regulates protein stability. DISCUSSION/SIGNIFICANCE OF FINDINGS: We previously reported a role for DNA-PK(cs) in immunomodulation. We now have evidence that this occurs in part through stabilization of Egr1. We believe this novel finding will lead to uncovering a broader role for DNA-PK(cs) as a mediator of protein stability in T cells and provide support for targeting DNA-PK(cs) in immunosuppression therapy.

